# Laboratory and field evaluation of an entomopathogenic fungus, Isaria cateniannulata strain 08XS‐1, against Tetranychus urticae (Koch)

**DOI:** 10.1002/ps.4233

**Published:** 2016-02-26

**Authors:** Xiaona Zhang, Daochao Jin, Xiao Zou, Jianjun Guo

**Affiliations:** ^1^ Institute of Entomology, the Provincial Key Laboratory for Mountainous Region Agricultural Pest ManagementGuizhou UniversityGuiyangChina; ^2^ Institute of Fungal ResourcesGuizhou UniversityGuiyangChina

**Keywords:** Tetranychus urticae, Isaria cateniannulata, entomopathogenic fungus, infection, field control

## Abstract

**BACKGROUND:**

The two‐spotted mite, Tetranychus urticae Koch, is one of the most serious mite pests of crops throughout the world. Biocontrol of the mite with fungal agents has long been paid much attention because of the development of insecticide resistance and the severe restriction of chemical pesticides. In this study, the efficacy of submerged conidia of the entomopathogenic fungus Isaria cateniannulata strain 08XS‐1 against T. urticae eggs, larvae and female adults was evaluated at different temperatures and humidity in the laboratory and under field conditions.

**RESULTS:**

The results showed that a suspension of 2 × 10^7^ submerged conidia mL ^−1^ caused the highest mortalities of mite eggs, larvae and females (100, 100 and 70% respectively) at 100% relative humidity and 25 °C in the laboratory. In the field experiments against the mites, a suspension of 2 × 10^8^ submerged conidia mL ^−1^ achieved significant efficiency – the relative control effects were 88.6, 83.8 and 83%, respectively, in cucumber, eggplant and bean fields after 10 days of treatment.

**CONCLUSION:**

The results suggest that the I. cateniannulata strain 08XS‐1 is a potential fungal agent, with acceptable production cost of conidia, against T. urticae in the field in an area such as southwestern China with higher air humidity. © 2016 The Authors. *Pest Management Science* published by John Wiley & Sons Ltd on behalf of Society of Chemical Industry.

## 1 INTRODUCTION

The two‐spotted spider mite *Tetranychus urticae* (Koch 1863) (Acari: Tetranychidae) is a cosmopolitan agricultural pest that damages more than 1100 types of crop, including horticulture plants and ornamental plants.^1^, ^2^ The mites suck plant juices with piercing/sucking mouthparts, which causes greying or yellowing and eventually necrotic spots on the leaves, leading to flower browning and petal withering, which resemble spray burns.^3^ *T. urticae* is known for its short life cycle, metagenesis and rapid development of resistance to chemical pesticides. Therefore, scientists have long paid attention to biological control of this organism.^4^, ^5^ Entomopathogenic fungi, or entomogenous fungi, are currently the most studied biological control agents of pests such as caterpillars (*Plutella xylostella*), mites (*Cenopalpus lineola*), aphids and nematodes,^6^ especially sucking‐mouthpart ones, because oral contact is not required, which makes them superior to other biopesticides.^7^

*Isaria cateniannulata* (Liang) Samson & Hywel‐Jones, *Paecilomyces cateniannulatus* (Liang, 1981),^8^ has been isolated from a variety of insects and verified as one of the most significant entomopathogenic fungi.^9^ Its distribution in the forest is second after *Beauveria bassiana*.^10^ We previously reported that this fungus displayed high pathogenicity towards female *T. urticae*. It could result in 100% mortality of the female mites, and its sporogenous structure could be observed on the mite body 3–5 days after treatment.^5^ However, previous studies have just confirmed that the fungus is lethal to the mite; the field application method and control effects remain to be evaluated. Therefore, the present study primarily focuses on verifying the appropriate infection conditions and concentrations of *I. cateniannulata* for controlling different stages of *T. urticae* in the laboratory and field. This study may provide valuable data for establishing *I. cateniannulata* as a biopesticide against mites.

## 2 MATERIALS AND METHODS

### 2.1 The mite Tetranychus urticae

*T. urticae* was obtained from the Institute of Entomology, Guizhou University, Guiyang, China. The mites were fed with the *Phaseolus vulgaris* plant at 25 ± 1 °C and 75 ± 5% relative humidity (RH) under a 12:12 h L:D photoperiod in an artificial climate incubator. Female adults, larvae and eggs were used as test objects in laboratory bioassays owing to their being key control targets in practice.

All bioassays were conducted in a 9 cm mite petri dish with a rearing platform consisting of a piece of sponge (1 cm height) covered with a piece of black cloth and plastic wrap (4 cm^2^). An appropriate amount of water was added to the dish to moisten the sponge fully.

To obtain *T. urticae* eggs at uniform age, 30 mated females were arbitrarily taken from the dish and transferred onto a detached leaf whose petiole was twined with degreased cotton and placed in a petri dish (9 cm diameter). The females were allowed to lay eggs freely for 18–24 h and were then removed, and 40–60 eggs leaf^−1^ were left for use.

To obtain *T. urticae* larva at a uniform age, the above eggs were reared in mite petri dishes in the incubator (25 ± 1 °C, 12:12 h L:D) for approximately 5–6 days. Thirty larvae were used for the bioassay.

Uniform *T. urticae* females were prepared from the experimental mite population, which were derived from the uniform eggs and cultured in the incubator (25 ± 1 °C, 12:12 h L:D) for 10–12 days. Thirty adult females were used for the bioassay.

### 2.2 The fungus Isaria cateniannulata and pathogenicity tests

*I. cateniannulata* was obtained from the Institute of Fungal Resources, Guizhou University, Guiyang, China. The fungus was cultured in a fluid medium consisting of potato (200 g), glucose (20 g) and water (1000 mL)^5^ at 27 °C for 10 days in a biochemical incubator. The submerged conidia were obtained via filtration with two‐layer lens wiping paper. The concentrations of submerged conidia used for the assay were 2 × 10^4^, 2 × 10^5^, 2 × 10^6^, 2 × 10^7^ and 2 × 10^8^ submerged conidia mL^−1^. The controls were treated with water.

The *T. urticae* eggs, larvae and female adults were placed on the rearing platform and treated with 0.04 mL of each concentration of submerged conidia via the spraying method using a double‐tubed spray bottle (volume 40 mL) at a distance of 20 cm from the dish. A piece of fresh bean leaf (2 cm^2^) was placed on each platform and replaced with a new piece of leaf every other day.

Each mite stage was cultured with each concentration of conidia at 15, 20, 25, 30 and 35 °C and under specific relative humidity conditions (i.e. 75, 85, 95 and 100%), and all treatments were repeated 3 times. The relative humidity was regulated using the saturated solution method.^11^

The infection and mortality of the eggs, larvae and females were observed for 7 days, and observation was continued until the infected numbers or mortality rates were steady for 3 days. The unhatched eggs and dead mites (larvae and adults) were then observed for 2–3 days to confirm the infection by sporogenous structures and conidia of the fungi under an optical microscope.

### 2.3 Field control experiments of mites with fungus

The vegetables were cucumber (*Cucumis sativus* L.), bean (*Phaseolus vulgaris* L.) and eggplant (*Solanum melongena* L.). The cucumber variety was Zhong‐Nong No. 8 from the Chinese Academy of Agricultural Sciences, the bean variety was Jin‐Qian White from Guizhou Guiyang Golden Agricultural Science and Technology Co., Ltd, and the eggplant variety was Qian‐Qie No.3 from Guizhou Academy of Agricultural Sciences.

Field experiments were conducted at the Teaching Experimental Farm (26° 24′ N, 106° 40′ E, 1131 m a.s.l.) of Guizhou University from 18 to 30 July 2013 (the middle growth stages of the vegetables). The experimental fields were not treated with any chemical pesticides, and routine irrigation (3 times before the experiment) and fertilisation (2 times before the experiment) were used in the fields. Each treatment was repeated 3 times (three plots), and each plot consisted of 15 vegetable plants, with 45 plots in total. The vegetables were grouped into rows, and each row was approximately 15 m^2^. The treatments were applied in a completely randomised block design. After inoculation with mites for 5–7 days, the adaxial surface of the leaves of the plants in each plot was treated with a fungal solution (1 L every row) containing 2 × 10^8^ submerged conidia mL^−1^ using an intelligent electric sprayer (MH‐D 16–3; Meng Hua Sprayer Co., Ltd, Taizhou, Zhejiang, China). The untreated (CK) plot was treated with water.

Prior to treatment with the fungal solution, ten sample plants were selected randomly from each plot. Eight leaves from each sample plant, one leaf in every direction (i.e. southward, eastward, northward and westward) at the middle and headpiece, were examined to calculate the initial mite population, including adults, nymphs and larvae. After treatment, sample leaves were prepared using the same method and transported to the laboratory for examination of mite numbers by stereoscopic microscope every day (24 h). The survey continued for 10 days. Local climate data were obtained from the local weather station during the field survey (Table 1).

**Table 1:** Local weather data during the field experiment (18–30 July 2013)

Date (days)	Weather	Temperature (°C)	Humidity (%)	Date (days)	Weather	Temperature (°C)	Humidity (%)
18	Cloudy	18–25	73–92	24	Cloudy	22–29	78–92
19	Light rain	18–22	92–99	25	Cloudy	22–29	50–90
20	Cloudy	22–29	56–90	26	Cloudy	22–33	52–89
21	Cloudy	22–30	76–98	27	Cloudy	22–30	54–85
22	Cloudy	22–29	64–92	28	Rain shower	22–29	82–89
23	Cloudy	22–32	61–86	29	Cloudy	22–29	74–95

### 2.4 Data analysis

The data were calculated using Excel 2007, SPSS v.17.0 and Origin85.

The parameters related to evaluation of the field control effect were as follows:

$$\begin{array}{cc} & \text{Corrected mortality rate}\;\left(\%\right)\\ & \;=\frac{\begin{array}{c} \text{test mites}-\text{medication treatment}\\ \text{group average survival}+\text{water treatment}\\ \text{average number of deaths} \end{array}}{\text{test mite number}}\times 100\% \end{array}$$

$$\begin{array}{cc} & \text{Population decrease rate}\;\left(\%\right)\\ & \;=\frac{\begin{array}{c} \text{population prior}\;to\;\text{treatment}\\ -\;\text{population after treatment} \end{array}}{\text{population prior}\;to\;\text{treatment}}\times 100\% \end{array}$$

$$\begin{array}{cc} & \text{Control efficiency}\;\left(\%\right)\\ & \;=\frac{\begin{array}{c} 1-\text{population without treatment}\\ \times \;\text{treatment population} \end{array}}{\begin{array}{c} \text{treatment population}\\ \times \;\text{population without treatment} \end{array}}\times 100\% \end{array}$$

The probit analytical method in SPSS software was used for general statistical analysis; chi‐square tests of the bioassay and field experiment data were used to obtain chi‐square values (*χ* ^2^) and linear regression equations regarding the relationship between the mite mortality risk value (*y*) and the number (*x*). When the results were less than *χ* ^2^ (3, 0.05) = 7.81, *P* > 0.05, there were no significant differences between the observed and theoretical values, and the concentration regression line was considered to agree with the practical values.

## 3 RESULTS

### 3.1 The mortality of mites infected with submerged conidia and the LC_50_ linear regression line

**Figure 1:**
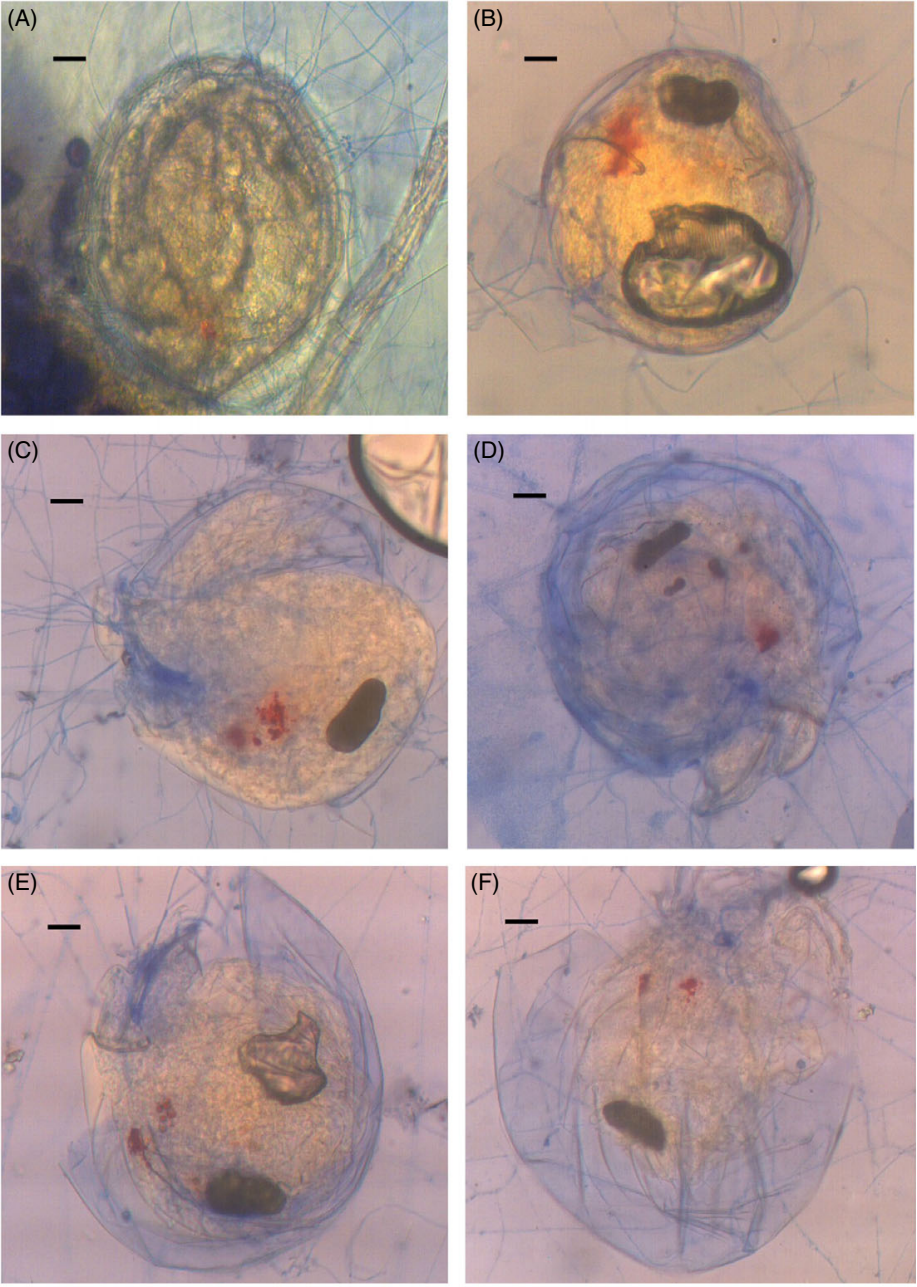
Embryonic development of T. urticae eggs infected by I. cateniannulata. A to F: embryonic development at 12, 24, 48, 96, 120 and 144 h after treatment with a submerged conidial suspension of I. cateniannulata. Scale bars: 20 µm.

The newly laid eggs of the mites were round, approximately 0.13 mm in diameter, shiny and transparent. It was difficult to observe the infection of the eggs during early infection by the naked eye or under a stereoscopic microscope because there were no obvious differences between the infected and uninfected eggs. At 3–7 days after treatment, mycelia and sporulation structures gradually appeared on the unhatched infected eggs (Fig. 1), and development of the fungus stabilised for approximately 10 days, whereas the unhatched eggs in the CK did not have mycelia. This suggests that infection by submerged conidia was lethal to mite eggs, although the embryo could develop with fungal growth (Fig. 1) under laboratory conditions.

The concentrations of submerged conidia caused high mortality rates in eggs, larvae and female adults under four humidity conditions at 15, 20, 25°, 30 and 35 °C. The regression equations and chi‐square test values, based on the mortality probability values (*y*) and the concentration logarithm values (*x*), are shown in Tables 2, 3, 4, 5, 6. All of the chi‐square test results were less than *χ* ^2^ (2, 0.05) = 5.99, *P* > 0.05, which indicates that there were no significant differences between the observed and theoretical values, i.e. the derived regression equation might conform to reality. The mortality observed in the three mite stages was positively associated with the concentration of submerged conidia and with the humidity at the specified temperature. The 2 × 10^7^ submerged conidia mL^−1^ caused the highest mortalities of mite eggs, larvae and females (100, 100 and 70% respectively) at optimum RH and optimum temperature.

**Table 2:** LC_50_ linear regression of the mortality of T. urticae infected with I. cateniannulata under various humidity conditions at 15 °C

Mite state	Humidity (%)	Regression equation (*y* =)	LC_50_ (submerged conidia mL^−1^) × 10^2^	95% CL	*χ* ^2^
Female	100	0.378*x* − 2.461	6.516	6.191–6.890	5.903
Female	95	0.389*x* − 3.032	7.788	7.366–8.408	4.613
Female	85	0.384*x* − 3.344	8.708	8.117–9.703	2.971
Female	75	0.483*x* − 4.575	9.467	8.736–10.939	5.686
Larva	100	0.315*x* − 1.479	4.694	4.278–5.014	3.888
Larva	95	0.245*x* − 1.492	6.095	5.747–6.454	2.581
Larva	85	0.260*x* − 1.968	7.559	7.151–8.140	3.130
Larva	75	0.316*x* − 3.199	10.122	9.296–11.530	2.662
Egg	100	0.383*x* − 3.670	9.594	8.963–10.597	4.572
Egg	95	0.391*x* − 3.968	10.135	9.353–11.494	4.069
Egg	85	0.409*x* − 4.201	10.271	9.446–11.760	5.346
Egg	75	0.400*x* − 4.454	11.132	9.933–13.817	4.267

**Table 3:** LC_50_ linear regression of the mortality of T. urticae infected with I. cateniannulata under various humidity conditions at 20 °C

Mite state	Humidity (%)	Regression equation (*y* =)	LC_50_ (submerged conidia mL^−1^) × 10^2^	95% CL	*χ* ^2^
Female	100	0.292*x* − 1.147	3.927	2.930–4.512	3.793
Female	95	0.290*x* − 1.371	4.723	4.026–5.189	3.287
Female	85	0.220*x* − 1.164	5.280	4.509–5.819	2.421
Female	75	0.297*x* − 1.892	6.368	5.964–6.832	2.416
Larva	100	0.919*x* − 3.833	4.169	3.984–4.321	4.295
Larva	95	0.673*x* − 3.145	4.674	4.486–4.839	4.655
Larva	85	0.585*x* − 3.267	5.585	5.420–5.744	3.060
Larva	75	0.530*x* − 3.494	6.588	6.413–6.776	3.252
Egg	100	0.353*x* − 3.157	8.942	8.430–9.705	2.701
Egg	95	0.352*x* − 3.311	9.395	8.788–10.344	4.560
Egg	85	0.333*x* − 3.477	10.436	9.530–12.043	2.847
Egg	75	0.377*x* − 4.216	11.181	9.977–13.784	3.436

**Table 4:** LC_50_ linear regression of the mortality of T. urticae infected with I. cateniannulata under different humidity conditions at 25 °C

Mite state	Humidity (%)	Regression equation (*y* =)	LC_50_ (submerged conidia mL^−1^) × 10^2^	95% CL	*χ* ^2^
Female	100	0.774*x* − 2.632	3.539	2.962–3.890	3.970
Female	94	0.744*x* − 2.896	3.895	3.481–4.181	4.705
Female	84	0.407*x* − 1.721	4.230	3.646–4.633	4.752
Female	75	0.470*x* − 2.789	5.937	5.664–6.208	5.019
Larva	100	0.663*x* − 1.960	3.097	2.522–3.472	5.815
Larva	94	0.449*x* − 1.403	3.123	2.498–3.554	5.537
Larva	84	0.449*x* − 2.467	5.493	5.278–5.692	4.421
Larva	75	0.310*x* − 1.929	6.226	5.952–6.516	4.593
Egg	100	0.397*x* − 2.703	6.805	6.574–7.071	2.832
Egg	94	0.385*x* − 2.974	7.734	7.426–8.136	3.119
Egg	84	0.417*x* − 3.677	8.820	8.381–9.455	3.725
Egg	75	0.446*x* − 4.540	10.174	9.385–11.616	3.387

**Table 5:** LC_50_ linear regression of the mortality of T. urticae infected with I. cateniannulata under different humidity conditions at 30 °C

Mite state	Humidity (%)	Regression equation (*y* =)	LC_50_ (submerged conidia mL^−1^) × 10^2^	95% CL	*χ* ^2^
Female	100	0.315*x* − 2.715	8.616	7.957–9.775	2.595
Female	97	0.363*x* − 3.273	9.013	8.327–10.233	3.081
Female	83	0.327*x* − 3.284	10.048	9.004–12.296	3.298
Female	75	0.318*x* − 3.419	10.751	9.419–14.079	5.205
Larva	100	0.539*x* − 3.878	7.196	7.002–7.420	3.598
Larva	97	0.383*x* − 3.414	8.921	8.439–9.630	2.544
Larva	83	0.515*x* − 4.982	9.677	9.061–10.746	4.601
Larva	75	0.615*x* − 6.111	9.938	9.196–11.566	3.277
Egg	100	0.432*x* − 24.086	9.463	8.893–10.361	4.370
Egg	97	0.403*x* − 3.994	9.899	9.193–11.087	4.843
Egg	83	0.463*x* − 4.929	10.638	9.632–12.842	3.375
Egg	75	0.508*x* − 5.700	11.212	9.769–16.632	3.733

**Table 6:** LC_50_ linear regression of the mortality of T. urticae infected with I. cateniannulata under different humidity conditions at 35 °C

Mite state	Humidity (%)	Regression equation (*y* =)	LC_50_ (submerged conidia mL^−1^) × 10^2^	95% CL	*χ* ^2^
Female	100	0.363*x* − 3.679	10.147	9.106–12.434	4.299
Female	97	0.376*x* − 4.152	11.057	9.590–15.476	3.430
Female	83	0.481*x* − 5.203	10.824	9.399–16.715	4.768
Female	75	0.383*x* − 4.677	12.202	9.899–31.904	3.974
Larva	100	0.567*x* − 5.417	9.561	8.988–10.565	4.123
Larva	97	0.594*x* − 5.853	9.848	9.153–11.260	2.312
Larva	83	0.558*x* − 5.897	10.568	9.503–13.562	3.110
Larva	75	0.745*x* − 7.436	9.979	9.115–12.922	3.557
Egg	100	0.432*x* − 4.297	9.948	9.237–11.172	3.214
Egg	97	0.415*x* − 4.362	10.506	9.586–12.280	5.072
Egg	83	0.405*x* − 4.736	11.685	10.139–16.176	3.027
Egg	75	0.724*x* − 8.003	11.060	—	2.987

### 3.2 The mortality of mites at different temperatures

**Figure 2:**
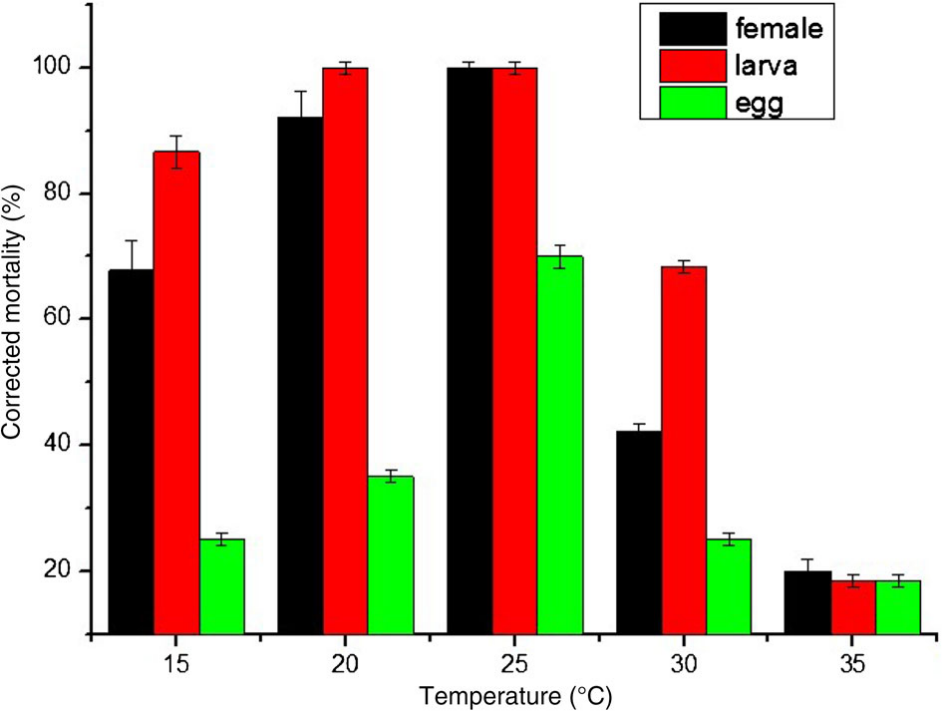
Mortality of T. urticae infected with a suspension of 2 × 10^8^ submerged conidia mL^−1^ at different temperatures and 100% humidity.

Figure 2 shows that there was a tendency for mite mortality to increase with humidity, and that it was highest in the suitable temperature range for the fungus growth and when the humidity was 100%; the mortality of *T. urticae* infected with *I. cateniannulata* increased at temperatures below 25 °C and decreased at temperatures above 25 °C. Therefore, *I. cateniannulata* prefers high humidity and low temperature. Obvious differences were found between the mortality rates of the three mite states within the temperature gradient, i.e. the mortality rates of the larvae and female adults were significantly higher than the mortality rates of the eggs. The highest mortality rates for all three states were observed at 25°C.

### 3.3 Effect of I. cateniannulata infection against T. urticae in the field experiments

The mite population densities on the cucumbers in the treated and CK fields showed no difference (*F* = 0.088, *P* = 0.782) before treatment. After 2 days of treatment, the mite population density decreased in the treated fields and was significantly different from the population density in the CK field (*F* = 57.563, *P* < 0.01). Before treatment, the mite population densities in eggplant fields showed no significant difference (*F* = 0.036, *P* = 0.859). After 2 days of treatment, the mite population density decreased in the treated field and there was significant difference between the treatment group and CK (*F* = 44.161, *P* < 0.01). The mite population densities in the treated and CK bean field groups showed no significant difference (*F* = 0.127, *P* = 0.740). After 2 days of treatment, the population density decreased in the treated field and was significantly different from the CK group (*F* = 45.000, *P* < 0.01). Table 7 shows that the mite population density gradually decreased with increasing time in the three vegetable fields treated with the fungal agent, and these population densities were significantly different from those of the CK groups.

**Table 7:** Population densities of T. urticae in three vegetable fields treated with submerged conidial suspension of I. cateniannulata a

Treatment	Live population of *T. urticae* (mites leaf^−1^)					
	0 days	2 days	4 days	6 days	8 days	10 days
Cucumber	124.333 ± 3.844 a	106.333 ± 3.179 b	64.000 ± 2.516 c	30.333 ± 0.881 d	22.000 ± 1.154 e	18.000 ± 0.577 e
CK1	128.333 ± 1.667 c	132.000 ± 1.154 c	145.333 ± 2.905 b	147.333 ± 1.452 b	168.000 ± 1.527 a	161.333 ± 3.527 a
*F*‐test	*F* = 0.088, *P* = 0.782	*F* = 57.563, *P* < 0.01	*F* = 447.639, *P* < 0.01	*F* = 4738.500, *P* < 0.01	*F* = 5813.455, *P* < 0.01	*F* = 1607.826, *P* < 0.01
Eggplant	123.667 ± 0.881 a	103.667 ± 1.855 b	73.000 ± 3.511 c	66.000 ± 0.577 d	44.000 ± 0.577 e	22.000 ± 0.577 f
CK2	128.333 ± 1.667 c	132.000 ± 1.154 c	145.333 ± 2.905 b	147.333 ± 1.452 b	168.000 ± 1.527 a	161.333 ± 3.527 a
*F*‐test	*F* = 0.036, *P* = 0.859	*F* = 44.161, *P* < 0.01	*F* = 177.817, *P* < 0.01	*F* = 4873.500, *P* < 0.01	*F* = 485.645, *P* < 0.01	*F* = 1062.316, *P* < 0.01
Bean	124.333 ± 1.452 a	116.667 ± 0.881 b	89.667 ± 0.881 c	56.000 ± 1.527 d	32.000 ± 0.577 e	25.667 ± 1.855 f
CK3	125.333 ± 2.403 d	126.667 ± 1.201 d	132.333 ± 1.201 c	137.333 ± 0.881 b	148.333 ± 0.881 a	151.667 ± 1.667 a
*F*‐test	*F* = 0.127, *P* = 0.740	*F* = 45.000, *P* < 0.01	*F* = 819.200, *P* < 0.01	*F* = 2126.286, *P* < 0.01	*F* = 12180.100, *P* < 0.01	*F* = 2551.500, *P* < 0.01

a The data are presented as the mean ± SE. The same letters in the same column represent no significant differences between the groups at the *P* = 0.05 level by Duncan's multiple range Test (DMRT).

The relative control effects of *I. cateniannulata* on *T. urticae* are shown in Table 8. At 10 days after treatment, the relative control efficacy reached 88.6, 83.8 and 83%, and the mite population decline rates were 86.2, 83 and 82% in the treated cucumber, eggplant and bean fields respectively. All of the rates were significantly different from the rates of the CK groups (*P* < 0.01).

**Table 8:** The relative control efficacy and population decline rate of T. urticae after spraying with submerged conidial suspension of I. cateniannulata a

Treatment	Relative efficacy (%)			Population decline rate (%)		
	2 days	6 days	10 days	2 days	6 days	10 days
Cucumber	18.20 ± 2.41 c	79.1 ± 0.77 b	88.65 ± 0.51 a	18.66 ± 1.30 c	76.79 ± 0.43 b	86.22 ± 0.45 a
Eggplant	9.26 ± 1.92 c	45.51 ± 0.86 b	83.79 ± 0.71 a	20.24 ± 1.71 c	49.23 ± 0.21 b	83.07 ± 0.48 a
Bean	7.64 ± 1.23 c	59.12 ± 1.04 b	83.02 ± 1.28 a	10.47 ± 1.20 c	57.04 ± 0.93 b	80.26 ± 1.54 a
*F*‐test	*F* = 8.784, *P* < 0.01	*F* = 351.721, *P* < 0.01	*F* = 11.596, *P* < 0.01	*F* = 13.598, *P* < 0.01	*F* = 556.468, *P* < 0.01	*F* = 9.331, *P* < 0.01

a The data are presented as the mean ± SE. The same letters in the same column represent no significant differences between the groups at the *P* = 0.05 level by DMRT.

Based on the above studies, the *I. cateniannulata* field efficacy dose corresponding to an experimental spore concentration of 2 × 10^8^ submerged conidia mL^−1^ was 4.5 × 10^12^ submerged conidia ha^−1^, which showed effective control results against *T. urticae* after 10 days of treatment in vegetable fields.

## 4 DISCUSSION

Mite control programmes are now aimed at maintaining the *Tetranychus* population at low levels throughout the year, i.e. the populations are allowed to reach an acceptable level before the mites become difficult to control. Therefore, the important targets in integrated pest management (IPM) of the mites are the eggs and females. Some studies have indicated that the acaricidal activities of many entomopathogenic fungi towards *T. urticae* can be attributed to disruption of mite development through their penetration and subsequent nutrient taking.^12^, ^13^ The results from a study by Zhang *et al.* 12 showed that the ovicidal activities of both *B. bassiana* and *I. fumosorosea* towards *T. urticae* eggs can be attributed to disruption of embryo development. Shi and Feng^13^ show that infection by *B. bassiana*, *I. fumosorosea* or *M. anisopliae* not only kills *T. urticae* females but also greatly reduces their fecundity. We obtained similar results: the infection of *I. cateniannulata* not only killed *T. urticae* females but also disrupted the development of the embryos. Shi^14^ found that mite eggs crinkled slightly in the early infection stage from *Beauveria bassiana*, *P. fumosoroseus* or *M. anisopliae*. At 1 week after treatment with high spore concentrations of these fungi, the unhatched eggs were heavily deformed and shrunken. In the present study, the mite eggs infected with *I. cateniannulata* did not become smaller and lighter, and the embryo was able to develop, which may be due to the fungus growing relatively slowly and the infected eggs being able to maintain their lives in the early infection days. However, the eggs finally died during the fungal infection process. Death might be caused by substances in the mite eggshells and associated with the infection mechanism of the fungus. However, this hypothesis requires further study.

*I. cateniannulata* is exclusively distributed throughout Asia^6^ but mostly found in China and Japan. The fungus has been used to control *Cenopalpus lineola*,^10^ and its pathogenicity towards *T. urticae* has recently been reported.^5^ The present study further demonstrates, via laboratory and field experiments, that the *I. cateniannulata* 08XS‐1 strain might be an effective biocontrol agent against *T. urticae*.

The results from this study showed that entomopathogenic fungal product *I. cateniannulata* strain 08XS‐1 was effective against eggs, larvae and females of *T. urticae*. The mite pathogens could be used as a main factor in the IPM strategy to enhance control efficiency of the two‐spotted mite, especially against the initial mite source. The results of this study also indicate that the larval stage is the most susceptible to the 08XS‐1 product. However, these results were not consistent with those reported for *T. urticae* infected with *B. bassiana* 15 or for *T. evansi* infected with *B. bassiana* or *M. anisopliae*.^16^ Therefore, such applications should be made at a time when most individuals in the mite population are at a susceptible stage of development and with an appropriate fungal strain. The eggs were more resistant than the other developmental stages to the fungus, and similar results were reported for eggs infected by *B. bassiana*.^15^ The reasons for this may include the eggshell not being suitable for the establishment of conidia because of its surface topography^17^ and/or there being less oxygen for the development of the fungus and the development of the embryo in the egg.^12^

Most entomopathogenic fungi are generally used when total eradication of a pest is not required. Instead, insect populations are controlled below an economic threshold, with some crop damage being acceptable.^18^ In addition, under the current protocols for producing mycoinsecticides, if the field application concentration dose does not exceed 5 × 10^13^ submerged conidia ha^−1^, the cost of production is acceptable.^19^ Therefore, *I. cateniannulata* has the potential for biological control of *T. urticae* in the field and might be valuable as a commercial antimite fungus. However, success with entomopathogenic fungi is often based on considerable multidisciplinary financial investment in research and development from industry, aid agencies and governments. When commercial interests are absent, especially in the development of classical, inoculative and conservation strategies, then long‐term support from government is essential.^20^ Entomopathogenic fungi have considerable potential to become major components in sustainable IPM if there is continued investment in research, technology transfer and education.^18^

A number of entomopathogenic fungi have been evaluated for the control of *T. urticae*, including *B. bassiana*, *M. anisopliae*, *Verticillium lecanii* and *Hirsutella thompsonii*.^12^, ^13^, ^18^, ^20^, ^21^, ^22^, ^23^ A review of the literature concerning these indicates that comparatively little work has been conducted on the effect of *I. cateniannula* against *T. urticae*. Zhang *et al.* 5, 24 first reported that the infection of *I. cateniannulata* not only killed *T. urticae* females but also resulted in high mortality. They also reported that the fungus is not pathogenic to *Euseius nicholsi*, a predatory mite species of the pest mite.

The growth, sporulation, infectivity and survival of entomopathogenic fungi are greatly affected by temperature, RH and solar radiation.^21^, ^25^ Despite the night‐time temperatures being generally not in the suitable range for infection by *I. cateniannula*, a temperature of 22–29 °C and an RH of 68–91% (Table 1) in the daytime were optimal for infection of *T. urticae* by the 08XS‐1 fungal product during the field trials. The tolerance of fungal isolates to varying temperature in a target agricultural ecosystem is essential if they are to be used in pest management programmes.^26^ Because temperature affects the physiology of the fungus and host, as well as the ability of the fungus to infect the host, the temperature tolerance of the fungi should be considered in any biological control programme.^27^, ^28^ Another principal factor limiting the viability of entomopathogenic fungi is the inactivation of conidia by ultraviolet radiation.^25^ We performed spraying of the fungus suspension on ten cloudy days, and therefore the role of ultraviolet radiation could not be considered. However, our results indicate that high humidity (≥85%) after treatment is more essential than the daytime air temperature to the efficacy of the 08XS‐1 product (Fig. 1; Tables 7 and 8). The region where the field experiments were conducted has a climate characterised by moderate temperature and high humidity in July (Table 1). The key factor limiting the activity of the fungus is therefore self‐inactivation of conidia. The spores in mycoinsecticides generally produced by liquid fermentation have a thin wall and are easily inactivated.^29^ Furthermore, *Isaria* fungi require appropriate conditions and time to colonise, spread and reproduce in the field and distribute evenly in the air.^30^, ^31^ Therefore, we used liquid medium containing submerged conidia directly for bioassays and field experiments. The liquid medium could provide nutritional elements for the colonisation and reproduction of the fungus, which might be important factors enhancing its control effect on the mites.

Based on the results of our study, it can be concluded that the *I. cateniannulata* strain 08XS‐1 product may be used as a potential fungal agent to control *T. urticae* at 68–91% relative humidity and 22–29 °C in the field. Improvement in the control of *T. urticae* with entomopathogenic fungi can be expected with the testing of other fungal species and strains, more effective and conventional spraying and new formulations.
